# ESR1 mutation as an emerging clinical biomarker in metastatic hormone receptor-positive breast cancer

**DOI:** 10.1186/s13058-021-01462-3

**Published:** 2021-08-15

**Authors:** Jamie O. Brett, Laura M. Spring, Aditya Bardia, Seth A. Wander

**Affiliations:** 1 Department of Medicine, Massachusetts General Hospital, Boston, MA USA; 2 Harvard Medical School, Boston, MA USA; 3 Department of Medical Oncology, Massachusetts General Hospital Cancer Center, 55 Fruit Street, Boston, MA 02114 USA

**Keywords:** Breast cancer, Hormone receptor/estrogen receptor, ESR1 mutation, Resistance, Combination, SERD, SERM, SERCA, CDK4/6

## Abstract

In metastatic hormone receptor-positive breast cancer, ESR1 mutations are a common cause of acquired resistance to the backbone of therapy, estrogen deprivation by aromatase inhibition. How these mutations affect tumor sensitivity to established and novel therapies are active areas of research. These therapies include estrogen receptor-targeting agents, such as selective estrogen receptor modulators, covalent antagonists, and degraders (including tamoxifen, fulvestrant, and novel agents), and combination therapies, such as endocrine therapy plus CDK4/6, PI3K, or mTORC1 inhibition. In this review, we summarize existing knowledge surrounding the mechanisms of action of ESR1 mutations and roles in resistance to aromatase inhibition. We then analyze the recent literature on how ESR1 mutations affect outcomes in estrogen receptor-targeting and combination therapies. For estrogen receptor-targeting therapies such as tamoxifen and fulvestrant, ESR1 mutations cause relative resistance in vitro but do not clearly lead to resistance in patients, making novel agents in this category promising. Regarding combination therapies, ESR1 mutations nullify any aromatase inhibitor component of the combination. Thus, combinations using endocrine alternatives to aromatase inhibition, or combinations where the non-endocrine component is efficacious as monotherapy, are still effective against ESR1 mutations. These results emphasize the importance of investigating combinatorial resistance, challenging as these efforts are. We also discuss future directions and open questions, such as studying the differences among distinct ESR1 mutations, asking how to adjust clinical decisions based on molecular surveillance testing, and developing novel therapies that are effective against ESR1 mutations.

## Background and overview

For patients with hormone receptor (HR)-positive advanced breast cancer, resistance to endocrine therapy (ET) is an inflection point. Although most HR-positive breast cancers benefit from first-line ET, most eventually become endocrine-resistant. Second-line ET monotherapy has a median progression-free survival (PFS) of only 2–6 months, compared to 1–4 years for first-line ET [1]. A key mechanism of endocrine resistance is mutation of the ligand-binding domain (LBD) of Estrogen Receptor 1 (ESR1) encoding estrogen receptor α (ER), which has been intensely studied over the past decade, including efforts to elucidate biochemical and molecular effects, role in selection of appropriate treatment, and therapeutic vulnerabilities [2].

ESR1 mutations were discovered in breast cancer in 1997 [3]. However, the significant role of ESR1 mutations in ET resistance was not established until 2013, with genomic sequencing of metastatic breast cancer (MBC) [4–8]. Work published shortly thereafter on patient-derived circulating tumor cell (CTC) lines confirmed that primary cancer cells with ESR1 mutations are relatively resistant to endocrine therapy and sensitive to ESR1 depletion [9]. ESR1 mutations were previously not apparent in The Cancer Genome Atlas due to sequencing in this database of primary, treatment-naïve tumors [10]. This contrasts with treatment-naïve endometrial cancers where ESR1 mutations were found in 4% of tumors in the database [11]. Thus, ESR1 mutations highlight that while sequencing primary tumors is useful for identifying mechanisms of primary oncogenesis, sequencing metastatic and resistant tumors is crucial for identifying molecular mediators of disease progression.

The prevalence of ESR1 mutations in patients depends on prior duration and setting of endocrine therapy. Approximately 20–40% of patients who have received aromatase inhibition (AI) for MBC have ESR1 mutations, with prevalence varying by sites of metastatic disease [2, 5–7, 12–14]. In contrast, ESR1 mutation prevalence is only 4–5% in recurrent breast cancer after prior adjuvant AI (including recurrence while on adjuvant AI) [12, 14, 15], 1.5–7% after neoadjuvant AI [16, 17], and less than 1% in ET-naïve MBC [7, 10, 14]. Thus, ESR1 mutations in HR-positive breast cancer occur almost exclusively after AI in the metastatic setting.

ESR1 mutations alone, however, only partly account for endocrine resistance in MBC. About 50% of endocrine resistance cases are associated with an ESR1 mutation; other mechanisms, increasingly uncovered, include alterations in the PI3K-AKT-mTORC1, RAS-MAPK, and CDK4/6-RB-E2F pathways, and ESR1 loss, amplification, and translocation [18]. In addition, ESR1 mutations usually occur with several concurrent genomic alterations, and together, these confer a globally worse prognosis [19, 20]. Furthermore, current treatments include ET partnered with additional targeted therapy, such as inhibition of CDK4/6, PI3K, or mTORC1. In these situations, a general theme is that ESR1 mutation alone is insufficient for full resistance, although this remains to be modeled and studied experimentally.

**Fig. 1:**
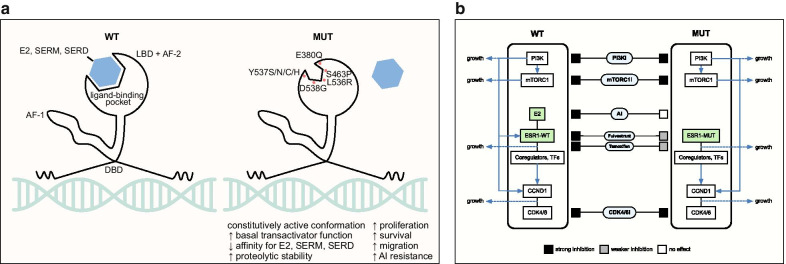
Mechanisms of resistance of ESR1 mutations. **a** Mutations and effects. All ESR1-MUT mutations are in the LBD. Mutations stabilize the active conformation in the absence of ligand, decreasing affinity for ligands, including estrogen, SERMs, and SERDs. This results in constitutive activity, increased basal activity, and proteolytic stability, enhancing cancer growth, metastasis, and resistance. E2: estradiol, AF-1: activation function 1 domain, LBD: ligand-binding domain, AF-2: activation function 2 domain, DBD: DNA-binding domain. **b** Key targeted pathways in HR-positive breast cancer and effects of ESR1-MUT. In the ESR1-WT situation, AI depletion of estrogen inhibits ESR1 activity, SERMs such as tamoxifen alter ESR1 binding partners and transactivation ability, and SERDs such as fulvestrant inhibit ESR1 activity and proteolytic stability. PI3Ki and mTORC1i inhibit upstream phospho-activation of ESR1 and additional growth-promoting signaling, and CDK4/6i inhibits the cell cycle machinery downstream of PI3K, mTORC1, and ESR1 signaling. In the ESR1-MUT situation, AI is ineffective since ESR1-MUT does not require estrogen, and tamoxifen and fulvestrant bind less strongly to ESR1-MUT (novel drugs in these categories are subject to ongoing study). PI3Ki and mTORC1i theoretically remain effective, although the crosstalk between ESR1-MUT and PI3K/mTORC1 signaling is not known. CDK4/6i is effective in both ESR1-WT and ESR1-MUT breast cancer. TF: transcription factor

There are many technologies for detecting ESR1 mutations in MBC. Sample sources include solid tissue biopsy, CTCs, and cell-free DNA (cfDNA); detection assays include next-generation sequencing (NGS) and droplet digital PCR (ddPCR), with ddPCR the most sensitive [21]. All ESR1 resistance mutations are in the LBD: the most common are D538G and Y537S; others include Y537N, Y537C, L536H, L536P, L536R, S463P, and E380Q [13, 22–25] (Fig. 1a). Based on cfDNA sampling in multiple studies, ESR1 mutations are polyclonal in a wide range of patients (20–70%) [12, 20, 26]. ESR1 fusions are rare but exhibit complete resistance to treatments targeting the LBD of ESR1 such as selective estrogen receptor modulators (SERMs) and degraders (SERDs), as these fusions lack the LBD [6, 27]. Thus, subclonality, polyclonality, and distinct effects of different ESR1 mutations demonstrate the utility inherent in liquid biopsy as compared to solid tissue sampling in characterizing the tumor ecosystem. On the other hand, liquid biopsy is subject to differences in CTC release or cfDNA shedding rates that may vary by tumor microenvironment and by the mutations themselves [28], making it important to continue comparing findings between liquid and solid tissue biopsies.

Prior reviews have discussed key conclusions from the literature on ESR1 mutations in MBC [2, 21–25]. Well-established findings are: (1) ESR1 mutations are acquired during AI treatment in the metastatic setting; (2) ESR1 mutations predict resistance to AI monotherapy; and (3) prospective studies on incorporating ESR1 mutation status in clinical decisions are lacking. Clinical practice is moving away from AI monotherapy for HR-positive MBC, however, as superior combination treatments have been demonstrated. Thus, this review will focus on how ESR1 mutations affect current care, including the rising use of ET in combination with targeted therapy and the use of existing and novel ER modulators, antagonists, and degraders.

## Mechanisms of resistance via ESR1 mutations

Due to rich foundational knowledge about ER structure and signaling and ongoing mechanism-based drug development, much is now known about the molecular consequences of ESR1 mutations [22] (Fig. 1).

The simplest mechanism by which ESR1 mutations produce resistance is constitutive activity. Whereas wild-type ESR1 (ESR1-WT) is bound by estrogen ligand to enable coactivator recruitment, LBD-mutated ESR1 (ESR1-MUT) is constitutively active and thus unaffected by AI depletion of estrogen [5, 7, 8, 29]. In the absence of ligand, compared to ESR1-WT, ESR1-MUT has increased stability of the active conformation, increased binding to coactivators, and decreased proteolytic degradation [5, 29–31].

In addition to constitutive activity, ESR1-MUT gains neomorphic and hypermorphic activity. ESR1-MUT transactivates altered sets of target genes, enhancing motility and metastasis [32–36]. ESR1-MUT has slightly altered interactomes, including increased association with FOXA1 and GREB1, although completely new partners have not yet been identified [35]. In addition, even in the presence of estrogen, ESR1-MUT can have far higher transactivation ability than ESR1-WT [4, 6, 7].

In pharmacokinetic assays, ESR1-MUT has relative resistance to standard ER-targeted therapies like tamoxifen and fulvestrant. For tamoxifen, ESR1-MUT has up to 30-fold decreased binding affinity (varying by specific mutation) and requires higher doses to inhibit transactivation function and cell proliferation [4, 5, 7, 8, 29, 31, 34]. For fulvestrant, ESR1-MUT has up to 40-fold decreased binding affinity and, like tamoxifen, requires an increased dose by an order of magnitude to inhibit transactivation function and cell expansion in culture and xenograft models [4, 5, 7, 8, 31, 34, 35, 37, 38].

Variegating these general resistance mechanisms of ESR1-MUT are mutation-specific and context-specific differences. For example, among the two most common mutations, Y537S compared to D538G has greater resistance to estrogen deprivation, tamoxifen, fulvestrant, and novel drugs like bazedoxifene and rintodestrant [5, 13, 30, 33, 34, 39]. On the other hand, D538G produces greater metastatic potential, especially to the liver, and unlike Y537S may increase Wnt signaling [8, 33, 40]. In addition, resistance of ESR1-MUT to tamoxifen and fulvestrant is far less prominent in HEK-293T cells compared to breast cancer cell lines [4], and ESR1-WT expression alongside ESR1-MUT reduces resistance [41]. This theme carries into the clinical data detailed below, where ESR1-MUT alone does not clearly confer resistance to tamoxifen or fulvestrant in patients.

In summary, conformational changes in ESR1-MUT decrease inhibitor binding, increase coactivator recruitment, and increase proteolytic stability to promote resistance to AI, tamoxifen, and fulvestrant in vitro. Altered target genes and context dependence, however, suggest opportunities to target and circumvent ESR1-MUT. Finally, the finding that higher doses of tamoxifen and fulvestrant are still effective and the characterization of the structure of ESR1-MUT have spurred development of novel ER-targeted molecules that may be highly effective at inhibiting ESR1-MUT.

## ESR1 mutations and aromatase inhibitors

ESR1-MUT clearly predicts poor response to single-agent AI, as previously reviewed [21, 24] (Table 1). For example, the SoFEA and EFECT trials of fulvestrant versus exemestane for patients with MBC after prior progression on non-steroidal AI were retrospectively analyzed for preexisting ESR1-MUT in cfDNA [12, 42]. ESR1-MUT, present at baseline in 39% of SoFEA and 23% of EFECT patients, predicted a significantly shorter PFS compared to ESR1-WT of 2.4 months versus 4.8 months, and a significantly lower one-year overall survival rate (OS) of 62% versus 79%.

**Table 1:** Studies analyzing how baseline ESR1 mutations affect clinical outcomes

Drugs	Study	Prior treatment	% MUT	Results	Conclusions
AI, SERD	SoFEA (NCT00253422, NCT00944918)	ET-resistant fulvestrant 0% chemo 0–1 lines	39	*PFS, SoFEA + EFECT* ESR1-WT exemestane 4.8 mo, fulvestrant 4.1 mo ESR1-MUT exemestane 2.4 mo, fulvestrant 3.9 mo (NS vs. WT) *1Y OS, SoFEA + EFECT* ESR1-WT exemestane 79%, fulvestrant 81% ESR1-MUT exemestane 62%, fulvestrant 80% (NS vs. WT)	ESR1-MUT does not predict response to fulvestrant
AI, SERD	EFECT (NCT00065325)	ET-resistant chemo 23%	23		
SERD	FERGI (NCT01437566)	ET-resistant fulvestrant 0% chemo 0–1 lines	37	*PFS* ESR1-WT fulvestrant 3.7 mo ESR1-MUT fulvestrant 3.5–7.4 mo (NS vs. WT)	ESR1-MUT does not predict response to fulvestrant
SERD	plasmaMATCH (NCT03182634)	ET-resistant (median 2 lines) chemo 66% CDK4/6i 10% mTORC1i 21%	100	*ORR* ESR1-MUT: 8% (12% if ESR1-MUT was the dominant clone) *PFS* ESR1-MUT: 2.2 mo	Heavily pretreated ESR1-MUT has short PFS on fulvestrant
SERD, CDK4/6i	PALOMA-3 (NCT01942135)	ET-resistant fulvestrant 0% chemo 34%	25	*ORR* ESR1-WT: fulvestrant 8.6%, fulvestrant + palbociclib 19% ESR1-MUT: fulvestrant 11.7%, fulvestrant + palbociclib 20.4% (NS vs. WT) *PFS* ESR1-WT: fulvestrant 5.4 mo, fulvestrant + palbociclib 9.5 mo ESR1-MUT: fulvestrant 3.6 mo, fulvestrant + palbociclib 9.4 mo (NS vs. WT)	ESR1-MUT does not predict response to fulvestrant + palbociclib
CDK4/6i	PEARL (NCT02028507)	ET-resistant fulvestrant 0% chemo 28%	29	*PFS* ESR1-WT: exemestane + palbociclib 9.3 mo ESR1-MUT: exemestane + palbociclib 5.7 mo (*p* = 0.06 vs. WT) ESR1-WT: fulvestrant + palbociclib 7.5 mo ESR1-MUT: fulvestrant + palbociclib 7.6 mo (NS vs. WT) *OS* ESR1-WT: exemestane + palbociclib 35 mo ESR1-MUT: exemestane + palbociclib 25 mo (* vs. WT) ESR1-WT: fulvestrant + palbociclib 30 mo ESR1-MUT: fulvestrant + palbociclib 27 mo (* vs. WT)	ESR1-MUT does not predict response to fulvestrant + palbociclib ESR1-MUT predicts resistance to exemestane + palbociclib
CDK4/6i	PADA-1 (NCT03079011)	∅	3.2	*PFS* ESR1-WT AI + palbociclib not reached ESR1-MUT AI + palbociclib 17.5 mo (* vs. WT)	ESR1-MUT predicts resistance to AI + palbociclib
CDK4/6i	nextMONARCH 1 (NCT02747004)	ET-resistant chemo 2+ lines CDK4/6i 0%	41	*PFS* ESR1-WT versus ESR1-MUT abemaciclib NS	ESR1-MUT does not predict response to abemaciclib
CDK4/6i, mTORC1i	TRINITI-1 (NCT02732119)	ET-resistant fulvestrant 37% chemo 8% CDK4/6i 100%	34	*PFS* ESR1-WT: exemestane + everolimus + ribociclib 6.9 mo ESR1-MUT: exemestane + everolimus + ribociclib 3.5 mo (* vs. WT)	ESR1-MUT predicts resistance to exemestane + everolimus + ribociclib
mTORC1i	BOLERO-2 (NCT00863655)	ET-resistant fulvestrant 17% chemo 26%	29	*PFS* ESR1-WT: exemestane 4.0 mo, everolimus + exemestane 8.5 mo ESR1-MUT: exemestane 2.8 mo, everolimus + exemestane 5.4 mo (* vs. WT)	ESR1-MUT predicts resistance to exemestane + everolimus
PI3Ki	NCT01870505	ET median 2 lines chemo median 2 lines	20*	*16-wk CBR* ESR1-WT: AI + alpelisib 62% ESR1-MUT: AI + alpelisib 0% (* vs. WT)	ESR1-MUT predicts resistance to AI + alpelisib

All studies except for PADA-1 were retrospective analyses of existing data. Sample sizes and references are in the main text. % MUT: percentage of patients in the analyzed cohort with baseline ESR1-MUT in cfDNA. *: solid sample, not cfDNA. Also notable was that 88% of patients had a baseline PIK3CA mutation in this study

In addition, serial cfDNA sampling revealed that ESR1-MUT develops months prior to radiologic progression on AI. In the plasmaDNA AI study, cfDNA was analyzed every three months for patients with MBC starting AI [20]. Of patients who progressed, 56% had ESR1-MUT at progression, and of these, 86% had ESR1-MUT detectable prior to progression at a median of 6.7 months beforehand. A similar effort from the FMER study found ESR1-MUT detectable in cfDNA prior to progression in 82% of patients at a median of 3.6 months beforehand [43]. The clinical utility of serial testing for ESR1 mutations, however, has not been demonstrated and is subject to evaluation in future trials. Furthermore, the field is moving away from AI monotherapy as first-line treatment for HR-positive MBC, and research has now focused on combination therapy and ER-targeting agents.

## ESR1 mutations and selective estrogen receptor modulators and antagonists

SERMs and selective estrogen receptor covalent antagonists (SERCAs) are promising drugs for MBC with ESR1-MUT. Although there is relative resistance of ESR1-MUT to tamoxifen in preclinical models, ESR1-MUT is not enriched in patients with prior tamoxifen monotherapy for MBC or even in patients with MBC resistant to tamoxifen [4, 7, 12, 24, 44]. This suggests the hypothesis, which remains untested, that ESR1-MUT does not cause tamoxifen resistance in the clinical setting, unlike in the preclinical setting.

Novel SERMs and SERCAs have been developed and tested specifically against ESR1-MUT (Table 2). Lasofoxifene, a SERM initially developed for osteoporosis and in that setting found to reduce breast cancer incidence [45], remarkably was found in vitro to retain efficacy in the presence of ESR1-MUT [41]. Lasofoxifene is currently in Phase 2 trials for patients with ESR1-MUT and for patients after progression on ET and CDK4/6 inhibition (CDK4/6i) (ELAINE: NCT03781063, ELAINE-2: NCT04432454). Bazedoxifene is a SERM/SERD hybrid that in addition to modulating co-regulator binding to ER also causes ER degradation, though interestingly only for WT and D538G but not for Y537S [30]. Bazedoxifene is more potent than tamoxifen and fulvestrant in ESR1-MUT breast cancer cells and is effective in tamoxifen-resistant cells and patient-derived xenograft (PDX) models [30, 46]. Already approved for use in postmenopausal hot flashes and osteoporosis, bazedoxifene is now in a Phase 2 trial for patients after progression on ET (NCT02448771). H3B-6545 is a drug optimized from the SERCA class that covalently inactivates ESR1 by targeting S530 [31] and in a Phase 1 study showed efficacy against ESR1-MUT MBC and against MBC previously treated with ET and CDK4/6i [47]. H3B-6545 is now in a Phase 2 trial for patients after progression on ET and CDK4/6i (NCT03250676).

**Table 2:** Novel SERM, SERCA, and SERD drugs targeting ER

Drug; Class	ESR1-MUT cells/PDX	Completed trials	Current trials
lasofoxifene; SERM	Drug effective; no resistance	PEARL Phase 3 trial for osteoporosis showed ↓ breast cancer incidence *Toxicities* arthralgia (25%), hot flashes (13%), VTE (1.5% over 5Y)	*Phase 2* NCT04432454 (ELAINE-2): lasofoxifene + abemaciclib for ESR1-MUT and progressed on ET NCT03781063 (ELAINE): lasofoxifene versus fulvestrant for ESR1-MUT and progressed on AI + CDK4/6i
bazedoxifene; SERM/SERD	Drug effective; relative resistance	FDA-approved, EMA-approved for postmenopausal osteoporosis/hot flashes *Toxicities* hot flashes (13%), arthralgia (11%), VTE (0.5% over 3Y)	*Phase 2* NCT02448771: bazedoxifene + palbociclib for progressed on ET
H3B-6545; SERCA	Drug effective; relative resistance	*Phase 1* NCT03250676: H3B-6545 progressed on ET + CDK4/6i: 47% stable disease, 9% partial response *Toxicities* Sinus bradycardia, diarrhea, nausea, fatigue, hot flashes, anemia	*Phase 1* NCT04288089: H3B-6545 + palbociclib for progressed on ET *Phase 2* NCT03250676: H3B-6545 for progressed on ET + CDK4/6i
Elacestrant (RAD1901); SERD	Drug effective; relative resistance	*Phase 1* NCT02338349: elacestrant progressed on fulvestrant and CDK4/6i: ORR 0%, 24-wk CBR 22%, PFS 1.9 mo progressed on ET: ORR 27%, 24-wk CBR 47%, PFS 5.4 mo *Toxicities* Nausea (33% G1-2), hypophosphatemia (25% G1-2, 8% G3), arthralgia (17%), fatigue (21% G1-2), diarrhea (12% G1-2), anemia (12% G1-2)	*Phase 3* NCT03778931 (EMERALD): elacestrant versus AI/fulvestrant for progressed on ET + CDK4/6i
Amcenestrant (SAR439859); SERD	Drug effective; relative resistance	*Phase 1/2* NCT03284957 (AMEERA-1): amcenestrant + palbociclib or alpelisib progressed on ET, ESR1-WT: 24-wk CBR 37% progressed on ET, ESR1-MUT: 24-wk CBR 32% *Toxicities* Nausea (18% G1-2), fatigue (18% G1-2), hot flashes (10% G1-2)	*Phase 2* NCT04059484 (AMEERA-3): amcenestrant versus AI/fulvestrant/tamoxifen for progressed on ET *Phase 3* NCT04478266 (AMEERA-5): amcenestrant + palbociclib versus letrozole + palbociclib for treatment-naïve
camizestrant (AZD9833); SERD	Drug effective; no resistance	*Phase 1* NCT03616587 (SERENA-1): camizestrant progressed on ET (82% fulvestrant, 68% CDK4/6i): ORR 14%, 24-wk CBR 67% *Toxicities* Visual disturbances (51% G1-2, 2% G3), sinus bradycardia (45% G1-2), nausea (18% G1-2), fatigue (13% G1-2), dizziness (8% G1-2, 2% G3)	*Phase 2* NCT04214288 (SERENA-2): camizestrant versus fulvestrant for progressed on ET NCT04588298 (SERENA-3): camizestrant versus fulvestrant for treatment-naïve *Phase 3* NCT04711252 (SERENA-4): camizestrant + palbociclib versus anastrozole + palbociclib for treatment-naïve
giredestrant (GDC-9545); SERD	Drug effective	*Phase 1* NCT03332797: giredestrant progressed on ET: ORR 11%, 24-wk CBR 44% *Toxicities* Fatigue (21% G1-2), nausea (21% G1-2), hot flashes (17% G1-2), arthralgia (17% G1-2), diarrhea (17% G1-2)	*Phase 2* NCT04576455 (acelERA): giredestrant versus fulvestrant/AI for progressed on ET *Phase 3* NCT04546009: giredestrant + palbociclib versus letrozole + palbociclib for treatment-naïve
rintodestrant (G1T48); SERD	Drug effective; no resistance	-	*Phase 1* NCT03455270: rintodestrant + palbociclib for progressed on ET
Zn-c5; SERD	Drug effective	-	*Phase 1* NCT04176747: ZN-c5 NCT04514159: ZN-c5 + abemaciclib NCT03560531: ZN-c5 + palbociclib
LSZ102; SERD	Not reported	-	*Phase 1* NCT02734615: LSZ102 + ribociclib or alpelisib for ET-resistant
ARV-471; SERD (PROTAC)	Drug effective	-	*Phase 2* NCT04072952: ARV-471 + palbociclib for progressed on ET
LY3484356; SERD	Not reported	-	*Phase 1* NCT04188548 (EMBER): LY3484356 + abemaciclib, everolimus, alpelisib, trastuzumab, AI in various combinations
D-0502; SERD	Drug effective	-	*Phase 1* NCT03471663: D-0502 + palbociclib for progressed on ET

Shown are preclinical data reporting efficacy against ESR1-MUT cells or PDX models, published trial results, and ongoing trials. References and details are in the main text

In summary, ESR1-MUT does not appear to be a main mechanism of resistance to tamoxifen, and the SERM lasofoxifene, SERM/SERD bazedoxifene, and SERCA H3B-6545 are all options in-development for endocrine-resistant and ESR1-MUT MBC.

## ESR1 mutations and selective estrogen receptor degraders

Despite relative resistance of ESR1-MUT to fulvestrant in pharmacokinetic studies in the laboratory, clinical studies show conflicting results regarding ESR1-MUT and response to fulvestrant (Table 1). In experiments, breast cancer cells expressing ESR1-MUT versus ESR1-WT require 10- to 50-fold higher doses of fulvestrant to achieve equivalent inhibition of ER transactivation function, cell proliferation, and PDX tumor growth [5, 7, 31, 34, 37, 38]. While this preclinical result is robust, it is not obvious how this translates to clinical consequences. There was concern that previous lower dosing of fulvestrant (250 mg) in patients was insufficient to inhibit ESR1-WT; current higher-dose regimens (500 mg) are more effective for ESR1-WT, but it is unknown whether they are adequate for ESR1-MUT [48]. This question has been studied in recent trials (detailed below), which may differ in results due to differences in concurrent mutations, prior lines of treatment, ESR1-MUT versus ESR1-WT relative expression, ESR1-MUT clonal prevalence, and ESR1 specific mutation types.

For example, the FERGI trial was retrospectively analyzed to determine how ESR1-MUT status affects response to fulvestrant [49]. Patients with MBC after progression on AI were randomized to fulvestrant versus fulvestrant plus pictilisib. Due to pictilisib toxicity, only the fulvestrant arm was analyzed for baseline ESR1-MUT effects, and sample sizes were small (40 patients with ESR1-WT, 30 patients with ESR1-MUT). ESR1-MUT prevalence did not increase on fulvestrant and did not predict resistance to fulvestrant: PFS was 3.7 months for ESR1-WT patients and 3.4–7.4 months for ESR1-MUT patients (varying by specific mutation, though limited by sample size). ESR1-MUT variant allele frequency (VAF) did not predict differential response to fulvestrant. Indeed, most patients with ESR1-MUT in the fulvestrant arm had decreasing VAF over time regardless of response or progression.

Similar results were obtained in the SoFEA and EFECT trials, which were discussed above regarding effects of ESR1-MUT in the AI arms [42]. Although ESR1-MUT predicted poor PFS and OS on exemestane, ESR1-MUT benefitted from fulvestrant, such that ESR1-MUT (73 patients) and ESR1-WT (147 patients) on fulvestrant had similar outcomes (PFS 3.9–4.1 months and one-year OS 79–81%). SoFEA was also analyzed separately rather than in combination with EFECT, with unchanged conclusions: on fulvestrant, PFS was 5.7 months for ESR1-MUT (23 patients) and 5.4 months for ESR1-WT (59 patients) [12].

In the PALOMA-3 trial, which will be discussed later regarding CDK4/6i, MBC patients after progression on AI were randomized to fulvestrant versus fulvestrant plus palbociclib. For the fulvestrant arm, PFS was 3.6 months for ESR1-MUT (28 patients) and 5.4 months for ESR1-WT (92 patients), and objective response rate (ORR) was 11.7% for ESR1-MUT and 8.6% for ESR1-WT, which were not significant differences [12]. Interestingly, however, there was a trend (*p* = 0.14) toward Y537S (19 patients) having the shortest PFS. In total, the FERGI, SoFEA, EFECT, and PALOMA-3 trials all show that general ESR1 mutation status does not predict worse response to fulvestrant.

However, recent data from the plasmaMATCH trial suggest that in the late-line setting, fulvestrant monotherapy may not be effective for patients with ESR1-MUT [50]. Patients with advanced breast cancer were matched to targeted therapies by cfDNA testing for mutations in ESR1, HER2, AKT, and PTEN. The population was heavily pretreated: patients had received a median of two prior lines of ET; 66% had also received prior chemotherapy, 10% prior CDK4/6i, and 21% prior mTORC1 inhibition. For the 74 patients with ESR1-MUT matched to receiving fulvestrant, PFS was just 2.2 months. Interestingly, of these patients, those with ESR1-MUT at VAF greater than 50% (44 patients) had ORR 12%, while patients with VAF less than 50% (30 patients) had ORR 0%. These data show that selecting heavily pretreated patients for fulvestrant based solely on the presence of ESR1-MUT is not an effective strategy. Tumors are heterogeneous and likely have multiple mechanisms of resistance beyond ESR1-MUT after having undergone multiple prior treatments.

Thus, novel oral SERDs should ultimately be developed for use in combination therapies, with the hope that these will be superior to fulvestrant for the ET backbone of the combination, especially for ESR1-MUT MBC (Table 2). Although ESR1-MUT for most novel SERDs has relative resistance similar to that for fulvestrant [38, 39, 51], these SERDs have excellent bioavailability, in contrast to fulvestrant [52]. In addition, some of these SERDs are effective preclinically against ESR1-MUT in cell lines and in PDX models that have complete fulvestrant resistance, through unknown mechanisms [38, 39, 53]. Elacestrant (RAD1901) has been effective against cells and PDX models with fulvestrant and CDK4/6i resistance [51], and Phase 1 data show efficacy in ESR1-MUT patients and patients pretreated with fulvestrant and CDK4/6i [54]. Elacestrant is in a Phase 3 trial for patients after progression on ET and CDK4/6i (EMERALD: NCT03778931). Amcenestrant (SAR439859) is effective against cells with ESR1-MUT and has shown efficacy in Phase 2 testing in patients with ESR1-MUT MBC [38, 55]. Amcenestrant is in a Phase 2 trial for patients after progression on ET (AMEERA-3: NCT04059484). Camizestrant (AZD9833) promisingly shows no relative resistance with ESR1-MUT Y537S in preclinical models [56], was effective against endocrine-resistant MBC in Phase 1 testing [57], and is in a Phase 2 trial for patients after progression on ET (SERENA-2: NCT04214288). Giredestrant (GDC-9545) showed clinical benefit in a Phase 1 study in patients after progression on fulvestrant and CDK4/6i [58] and is in a Phase 2 trial for patients after progression on ET (acelERA: NCT04576455). Side effect profiles so far are not substantially different from those of aromatase inhibitors, fulvestrant, and tamoxifen and include hot flashes, arthralgia, fatigue, upper and lower GI symptoms, and vision changes. There are numerous other SERDs in Phase 1 testing (Zn-c5, rintodestrant (G1T48), ARV-471, LSZ102, LY3484356, and D-0502) and even more in preclinical testing.

In summary, clinical data suggest that ESR1-MUT alone does not clearly confer fulvestrant resistance in patients, although the clinical experience has been mixed, and in preclinical models there is relative resistance requiring higher doses of fulvestrant for efficacy. In this area, novel oral SERDs with higher bioavailability will make SERD use more convenient and possibly more effective. However, ESR1-MUT develops primarily after exposure to AI and therefore comes with concurrent genetic and epigenetic resistance mechanisms to ET; thus, based on current evidence, the knowledge that a patient has ESR1-MUT should not routinely lead toward SERD monotherapy but rather toward combination therapy, which may incorporate a novel oral SERD, SERM, or SERCA as the ET backbone.

## ESR1 mutations and CDK4/6 inhibitors

Combination ET plus CDK4/6i (palbociclib, ribociclib, or abemaciclib) is currently the most effective first-line treatment for HR-positive MBC. In general, for these and for other combination therapies, ESR1-MUT nullifies the AI component of the combination. However, the final consequences of this are difficult to predict due to cooperativity and compensatory interplay between ER signaling and the other pathways being targeted, further complicated by neomorphic and hypermorphic ESR1-MUT activity. For ET plus CDK4/6i, the emerging pattern is that ESR1-MUT is primarily resistant to AI plus palbociclib/ribociclib (Table 1). ESR1-MUT does not cause resistance to *fulvestrant* plus CDK4/6i (since fulvestrant remains effective against ESR1-MUT, as above), nor does it cause resistance to AI plus *abemaciclib* (which among CDK4/6 inhibitors has been proven effective as monotherapy).

In theory, ESR1-MUT would not confer resistance to CDK4/6i, as ER is upstream of Cyclin D-CDK4/6 inactivation of RB and derepression of E2F activity [59]. Consistent with this, in preclinical work, endocrine-resistant and ESR1-MUT breast cancer cells retain palbociclib sensitivity, and PDX models with ESR1-MUT also remain sensitive to palbociclib and abemaciclib [33, 37, 60]. However, in patients, endocrine resistance is heterogeneous and includes not just ESR1-MUT but other mechanisms that also create CDK4/6i resistance. For instance, PDX models with ESR1-MUT that also carry RB1 loss are resistant to palbociclib [37]. Studies of combinatorial resistance are needed to guide selection of subsequent therapies for patients with progressive MBC.

Recent studies have shown that palbociclib combined with fulvestrant, but not with AI, is effective in patients with ESR1-MUT. In a cohort of patients with MBC on ET and CDK4/6i, solid tumor tissue whole-exome sequencing was performed to examine how genetic changes associate with sensitive and resistant phenotypes [61]. In these patients, ESR1-MUT was present in 0% (0/13) with sensitivity to AI plus palbociclib, 60% (3/5) with sensitivity to fulvestrant plus palbociclib, 27% (4/15) with resistance to AI plus palbociclib, and 25% (4/16) with resistance to fulvestrant plus palbociclib. These data, albeit limited by sample size, suggest that ESR1-MUT is compatible with sensitivity to fulvestrant plus palbociclib but not AI plus palbociclib. The PEARL trial randomized patients with MBC after progression on AI to capecitabine versus exemestane plus palbociclib or fulvestrant plus palbociclib – this second cohort was created after the field had learned how ESR1-MUT causes AI resistance [62]. For patients on fulvestrant plus palbociclib, baseline ESR1-MUT (38 patients) yielded the same PFS as baseline ESR1-WT (102 patients) of 7.5–7.6 months. This was in contrast with ESR1-MUT predicting shorter PFS for exemestane plus palbociclib: PFS was 5.7 months for ESR1-MUT (41 patients) versus 9.3 months for ESR1-WT (104 patients). These data suggest that ESR1-MUT eliminates the ET contribution for AI but not fulvestrant in combination therapy.

Analysis of the larger PALOMA-3 trial bolsters these findings that ESR1-MUT does not predict resistance to fulvestrant plus CDK4/6i. Patients with MBC after progression on AI were randomized to fulvestrant versus fulvestrant plus palbociclib. Retrospective analysis found that the 63 patients with baseline ESR1-MUT and the 177 patients with baseline ESR1-WT had similar outcomes, with PFS 9.4–9.5 months [12]. An interesting additional analysis was conducted on 195 patients with baseline and end-of-treatment (progression) cfDNA samples [26, 63]. Although total ESR1-MUT prevalence was unchanged between baseline and end of treatment regardless of arm (25–31%), there was a significant enrichment for Y537S prevalence at end of treatment (11%) compared to baseline (4%) that did not occur for other ESR1 mutations. In summary, ESR1-MUT in general does not confer resistance to fulvestrant plus CDK4/6i, but the effects of Y537S deserve further exploration.

No trial to date has shown the clinical utility of monitoring ESR1 mutation status. The ongoing PADA-1 trial is the first with this specific goal [64]. In PADA-1, patients with MBC naïve to treatment begin therapy with AI plus palbociclib and receive serial cfDNA assays for ESR1-MUT. If ESR1-MUT clonal presence rises and the patient has not progressed, the patient is randomized to continuing AI plus palbociclib versus changing to fulvestrant plus palbociclib. The study will evaluate PFS and treatment safety after randomization. Given that ESR1-MUT appears 3.6–6.7 months prior to radiologic progression on first-line AI in the plasmaDNA and FMER studies described above [20, 43] and that ESR1-MUT alone does not cause fulvestrant plus palbociclib resistance, one hypothesis is that changing to fulvestrant plus palbociclib after the development of ESR1-MUT will lengthen PFS. However, it is also possible that ESR1-MUT development under the selective pressure of AI plus palbociclib marks the evolution of concurrent resistance mechanisms to CDK4/6i and that more than simply changing the ET backbone will be required.

In contrast with palbociclib/ribociclib, abemaciclib has demonstrated clinical efficacy as a monotherapy and is the only CDK4/6i approved as a single agent. In the nextMONARCH 1 trial, patients with MBC after progression on ET and at least two lines of chemotherapy were randomized to abemaciclib (two different doses) versus tamoxifen plus abemaciclib. Retrospective analyses of the abemaciclib monotherapy arms examined baseline and acquired mutations in cfDNA. For many genes, baseline mutation correlated with shorter PFS (PIK3CA, TP53, FGFR1, MYC, NF1, EGFR, ERBB2, CCNE1), but notably this was not the case for ESR1 [65]. At end of treatment (progression), only 6% of patients had acquired ESR1-MUT; other acquired mutations were more common (TP53, EGFR, RB1, MYC, MET) [66]. A similar lack of acquired ESR1-MUT on abemaciclib was found in the MONARCH 3 trial through retrospective analysis [66]. In MONARCH 3, patients with advanced breast cancer naïve to systemic therapy were randomized to AI plus abemaciclib versus AI alone. At end of treatment (progression), the investigators analyzed acquired mutations in cfDNA. AI plus abemaciclib compared to AI alone had fewer mutations in ESR1 (17% versus 31%), contrasting with more mutations in RB1 (6% versus 0%), MYC (5% versus 0%), and AR (5% versus 0%). Together, these data suggest that most cases of progression on abemaciclib are not due to ESR1-MUT.

Finally, emerging data suggest that abemaciclib monotherapy can produce durable responses in pretreated patients, even in some who have previously received palbociclib [60]. Patients after progression on ET plus palbociclib/ribociclib with ESR1-MUT had responses lasting for at least 16 months on abemaciclib monotherapy, and on fulvestrant plus abemaciclib. This did not occur if prior resistance came with concurrent CDK4/6i resistance mutations such as in RB1, reflecting preclinical findings [37].

In total, these studies indicate that ESR1-MUT does not cause resistance to CDK4/6i alone nor to the combination of fulvestrant plus CDK4/6i but does blunt the efficacy of AI plus palbociclib/ribociclib due to AI resistance. The PADA-1 trial is the first of its design to assess whether ESR1-MUT monitoring can be useful for therapy decisions. Results are much anticipated, and further trials of similar design are warranted.

## ESR1 mutations and PI3K/mTORC1 inhibitors

The PI3K-AKT-mTORC1 pathway is frequently activated by direct mutation and by indirect crosstalk with other mechanisms in HR-positive MBC [67]. ET combined with PI3K inhibition (PI3Ki) or mTORC1 inhibition (mTORC1i) is now a standard second-line therapy. There are limited data on how ESR1-MUT affects these combinations, and thus, further analyses are needed (Table 1).

PI3K-AKT-mTORC1 acts parallel to, downstream of, and upstream of ER, including through ligand-independent activation of ER [67]. As a result, PI3Ki and mTORC1i can be effective in endocrine-resistant and ESR1-MUT breast cancer cells [35, 51, 68, 69]. On the other hand, increased ER activity is a possible mechanism of adaptive resistance to PI3Ki [70]. Although PI3Ki and mTORC1i each have some efficacy as monotherapy, combination with ET is synergistic [71–74]. This is consistent with the trial data below suggesting that ESR1-MUT blunts the response to AI plus PI3Ki/mTORC1i due to resistance to the ET backbone.

One Phase 2 study has been analyzed for how ESR1-MUT affects AI combined with PI3Ki (alpelisib) [75]. Patients with MBC regardless of prior treatment received AI plus alpelisib, and solid tissue biopsies were analyzed retrospectively for baseline mutations. The patients had received a median of two lines of prior ET and two lines of prior chemotherapy in the metastatic setting; 88% had PIK3CA mutations and 20% had ESR1 mutations in the solid tissue biopsies. Although sample sizes were small, the results were striking: the four-month clinical benefit for ESR1-MUT (6 patients) was 0%, compared to 62% for ESR1-WT (32 patients). Follow-up xenograft studies of genetically modified breast cancer cells recapitulated these results, showing that Y537S blunts the effect of alpelisib plus estrogen deprivation. Helpful additional analyses would include those using cfDNA samples (not just solid tissue biopsy) in this trial and analyses of trials using fulvestrant plus PI3Ki, such as SOLAR-1, BYLieve, and SANDPIPER.

AI combined with mTORC1i (everolimus) has similar results against ESR1-MUT. In the BOLERO-2 trial, patients with MBC after progression on AI were randomized to exemestane plus everolimus versus exemestane alone. In the exemestane plus everolimus arm, ESR1-MUT (95 patients) had shorter PFS than ESR1-WT (257 patients) of 5.4 months versus 8.5 months, though there was still a significant benefit of everolimus addition compared to exemestane alone [19]. Similar results were reported in the TRINITI-1 Phase 2 trial [76]. In this study, patients with advanced breast cancer who had progressed on ET and CDK4/6i were treated with a triplet combination of exemestane, everolimus, and continuous ribociclib. There were no new toxicities compared to studies of two-drug combinations; common adverse events included neutropenia (69%; 51% grade 3/4), stomatitis (40%; 3% grade 3/4), hyperglycemia (18%; 7% grade 3/4), and hypophosphatemia (19%; 6% grade 3/4). PFS was shorter for ESR1-MUT (3.5 months, 30 patients) compared to ESR1-WT (6.9 months, 59 patients). As for PI3Ki, it would be useful to analyze the effects of ESR1-MUT in trials testing tamoxifen/fulvestrant in combination with everolimus (TAMRAD, PrE0102, and MANTA) and in additional trial data of AI plus everolimus (BOLERO-6).

Overall, further investigation is needed on ESR1-MUT in ET plus PI3Ki/mTORC1i. Extrapolating from the ET plus CDK4/6i data, one hypothesis is that SERD/SERM/SERCA drugs would be superior to AI when combined with PI3Ki/mTORC1i for patients with ESR1-MUT. Promising data were reported in a PDX model resistant to tamoxifen and fulvestrant plus palbociclib with ESR1-E380Q and PIK3CA-E545K/E722K, in which tumor growth was inhibited by elacestrant and even more so with elacestrant combined with everolimus [51]. In general, modeling combinatorial resistance in experiments is challenging and requires thorough characterization of concurrent mutations, expression and activity levels beyond genetic changes, non-cell-autonomous interactions among clones, and a deep understanding of involved signaling networks [61, 77].

## Open questions and future directions

Many questions remain about how ESR1 mutations affect current and developing clinical practice (Table 3). One area of investigation that has arisen from existing analyses revolves around how distinct ESR1 mutations (D538G, Y537S, and others) differentially impact patterns of resistance. All ESR1 LBD mutations cause complete AI resistance; however, preclinical studies indicate Y537S has the highest transactivation activity and the greatest relative resistance to tamoxifen, fulvestrant, and some of the novel SERDs and SERMs (the SERCA H3B-6545 is subject to ongoing study). In patients, PALOMA-3 and SoFEA analyses suggested that Y537S had the shortest PFS on fulvestrant and—unique among ESR1 mutations—was enriched in patients on fulvestrant and on fulvestrant plus palbociclib. Thus, one hypothesis may be that while all ESR1 LBD mutations are selected for in patients on AI monotherapy and predict poor response to AI, Y537S is the mutation driving resistance to ER-targeted therapies, and trials and analyses should stratify by mutation types present in each patient’s heterogeneous tumor. Larger sample sizes will be needed to test these ideas, and in this regard meta-analyses on distinct mutation effects could be helpful.

**Table 3:** Open questions and relevant ongoing trials

Open question	Ongoing trials
How do different ESR1 mutations (D538G, Y537S, others) differentially affect resistance?	NCT03250676: Phase 2 trial (~ 150 patients) of H3B-6545 for patients after progression on ET + CDK4/6i, with plan to analyze outcomes by different ESR1 mutations
How does selecting treatment based on the detection of ESR1-MUT in cfDNA affect clinical outcomes?	PADA-1 (NCT03079011): Phase 3 trial (~ 1000 patients) of randomizing patients on AI + palbociclib to continuing versus changing to fulvestrant + palbociclib after detection of ESR1-MUT in surveillance cfDNA ELAINE-2 (NCT04432454): Phase 2 trial (~ 25 patients) of lasofoxifene + abemaciclib for patients who progressed on ET and have ESR1-MUT ELAINE (NCT03781063): Phase 2 trial (~ 100 patients) of lasofoxifene versus fulvestrant for patients who progressed on AI + CDK4/6i and have ESR1-MUT
Are novel SERM/SERCA/SERD drugs superior to tamoxifen/fulvestrant for ESR1-MUT?	ELAINE (NCT03781063): Phase 2 trial (~ 100 patients) of lasofoxifene versus fulvestrant for patients who progressed on AI + CDK4/6i and have ESR1-MUT EMERALD (NCT03778931): Phase 3 trial (~ 500 patients) of elacestrant versus AI/fulvestrant for patients who progressed on ET + CDK4/6i AMEERA-3 (NCT04059484): Phase 2 trial (~ 400 patients) of amcenestrant versus AI/fulvestrant/tamoxifen for patients who progressed on ET SERENA-2 (NCT04214288): Phase 2 trial (~ 250 patients) of camizestrant versus fulvestrant for patients who progressed on ET acelERA (NCT04576455): Phase 2 trial (~ 300 patients) of giredestrant versus fulvestrant/AI for patients who progressed on ET
How can neomorphic/hypermorphic activities of ESR1-MUT be targeted?	
How does ESR1-MUT interact with PI3K-AKT-mTORC1 signaling?	
Why does AI in the adjuvant setting (as opposed to the metastatic setting) fail to select for ESR1-MUT?	
How does ESR1-MUT VAF reflect total tumor burden and progression?	
What are effective treatments for ESR1 fusions that lack the LBD?	
How can combinatorial resistance be modeled in experiments?	

Shown are questions and relevant trials discussed in the text. Additional preclinical questions are listed at the bottom

**Fig. 2:**
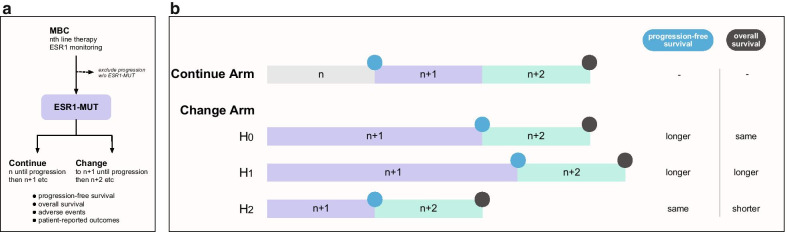
Trial design for testing the incorporation of ESR1 mutation monitoring into clinical decision-making. **a** Design. Patients with MBC start on standard treatment (such as AI plus CDK4/6i) with monitoring for the development of ESR1-MUT. Patients are excluded if they have clinical progression before ESR1-MUT arises (presumably due to development of other resistance mechanisms). Patients who develop ESR1-MUT are at that time randomized to continuing current therapy until progression, versus changing current therapy immediately. All patients change to next-line therapy at each progression. The main end points are OS, PFS, adverse events, and patient-reported outcomes. **b** Possible outcomes. Selection of endpoint is important: while for the “change” arm, longer PFS might be expected, OS has multiple plausible outcomes – (H0) no change in OS, due to the same clocklike rate of resistance development; (H1) longer OS, due to the “change” arm have a higher chance of durable response with earlier therapy switch; (H2) shorter OS, due to premature discontinuation of current therapy before attaining maximum benefit

Despite the predictive value of ESR1-MUT detection, the clinical utility of management decisions based on ESR1-MUT is unknown. In the plasmaMATCH trial, selection of extended-dose fulvestrant based solely on ESR1-MUT in heavily pretreated patients produced low response rates and PFS, likely due to concurrent genetic and epigenetic mechanisms of resistance. The ongoing PADA-1 trial is the first to specifically test incorporating ESR1-MUT monitoring into practice. As described above, PADA-1 will serially monitor patients on AI plus palbociclib for the development of ESR1-MUT and test whether switching the ET backbone based on this molecular alteration will improve outcomes. A key question is whether changing therapy based on ESR1-MUT detection before radiologic progression will improve long-term disease control and OS compared to simply changing therapy based upon radiologic progression. Further trials modeled after the PADA-1 design (Fig. 2) are needed in general to understand how to integrate cfDNA surveillance into a field that is heading toward ever more complex, precision-based combinations of targeted therapies.

Parallel to these investigations are studies focused on the development of novel therapeutic agents. The next generation of oral SERMs, SERCAs, and SERDs are promising treatments for any patient with HR-positive breast cancer. It will be important to understand how patients with ESR1-MUT fare on these therapies, as we hope that these will prove superior to tamoxifen and fulvestrant for these patients. An additional step will be testing these novel agents in the spirit of PADA-1, investigating the utility of cfDNA assays in therapy selection.

Finally, beyond directly targeting ER constitutive activity in ESR1-MUT cells, there may be additional vulnerabilities of ESR1-MUT tumors among the altered target genes, protein interactions, and downstream cellular adaptations. Examples include targeting IGF1R signaling [78], NOTCH signaling and cancer stem cell-like properties [32, 79], Wnt signaling [40], BCL2 [80], FOXA1 [32, 35], AR [36], CHI3L1 [36], TFAP2C [33], and histone acetyltransferases [33]. Further work is required to understand how ESR1-MUT may increase dependence on Cyclin D-CDK4/6, as preliminary PADA-1 data suggested clearance of ESR1-MUT on AI plus palbociclib in some patients [81]. Separate work on cfDNA in patients with MBC on various treatments showed that ESR1-MUT prevalence was depleted in patients who had received palbociclib (9%) compared to patients who had not (36%) [15]. This effect is not clearly seen in vitro and thus also raises the question of how ESR1-MUT might influence cancer cell vulnerabilities within the tumor environment.

## Conclusions

In summary, ESR1-MUT arises in patients who receive AI in the metastatic setting, and this causes resistance to AI monotherapy, with cfDNA detection of ESR1-MUT preceding radiologic progression by 3–7 months. In this review, we detail how ESR1-MUT influences response to therapies other than AI monotherapy in patients with MBC (Fig. 1b). ESR1-MUT breast cancer is likely still somewhat sensitive to tamoxifen and fulvestrant, although novel SERMs, SERCAs, and SERDs may improve efficacy further. ESR1-MUT does not cause CDK4/6i resistance, and emerging data suggest that abemaciclib monotherapy may still be an option for these patients. It is crucial to effectively model combination therapies as part of these translational research efforts. ESR1-MUT may selectively attenuate the efficacy of the anti-estrogen component of a combination regimen, and subsequent tumor response becomes dependent on sensitivity to the partner agent (CDK4/6i, PI3Ki, or mTORC1i). ESR1-MUT does blunt the efficacy of PI3Ki or mTORC1i in combination with AI due to nullification of the AI component, and thus, tamoxifen/fulvestrant in combination with PI3Ki or mTORC1i should be studied in the ESR1-MUT situation. Given the prevalence of ESR1-MUT, its intrinsic link with the processes driving HR-positive breast cancer, and its large impact on outcomes of a variety of therapies, ongoing trials should stratify patients by ESR1-MUT status. Future directions also include distinguishing among distinct ESR1 mutations, testing the utility of decision-making based on cfDNA surveillance, and directly targeting and circumventing ESR1-MUT with novel therapies.

## Data Availability

Not applicable.

## Abbreviations

AI: Aromatase inhibition
CBR: Clinical benefit rate
CDK4/6: Cyclin-dependent kinase 4 and cyclin-dependent kinase 6
CDK4/6i: Inhibitor of cyclin-dependent kinase 4 and cyclin-dependent kinase 6
cfDNA: Cell-free DNA
CTC: Circulating tumor cell
ddPCR: Droplet digital PCR
ER: Estrogen receptor α
ESR1: Estrogen receptor 1
ESR1-MUT: Tumor has ESR1 mutations
ESR1-WT: Tumor does not have ESR1 mutations
ET: Endocrine therapy
HR: Hormone receptor
LBD: Ligand-binding domain
MBC: Metastatic ER-positive breast cancer
mTORC1: Mammalian target of rapamycin complex 1
mTORC1i: Inhibitor of mammalian target of rapamycin complex 1
NGS: Next-generation sequencing
ORR: Objective response rate
OS: Overall survival
PDX: Patient-derived xenograft
PFS: Median progression-free survival
SERCA: Selective estrogen receptor covalent antagonist
SERD: Selective estrogen receptor degrader
SERM: Selective estrogen receptor modulator
VAF: Variant allele frequency
